# Bimodal distribution of RNA expression levels in human skeletal muscle tissue

**DOI:** 10.1186/1471-2164-12-98

**Published:** 2011-02-07

**Authors:** Clinton C Mason, Robert L Hanson, Vicky Ossowski, Li Bian, Leslie J Baier, Jonathan Krakoff, Clifton Bogardus

**Affiliations:** 1 From the Phoenix Epidemiology and Clinical Research Branch, National Institute of Diabetes and Digestive and Kidney Diseases, 1550 E. Indian School Rd., Phoenix, AZ, 85014, USA

## Abstract

**Background:**

Many human diseases and phenotypes are related to RNA expression, levels of which are influenced by a wide spectrum of genetic and exposure-related factors. In a large genome-wide study of muscle tissue expression, we found that some genes exhibited a bimodal distribution of RNA expression, in contrast to what is usually assumed in studies of a single healthy tissue. As bimodality has classically been considered a hallmark of genetic control, we assessed the genome-wide prevalence, cause, and association of this phenomenon with diabetes-related phenotypes in skeletal muscle tissue from 225 healthy Pima Indians using exon array expression chips.

**Results:**

Two independent batches of microarrays were used for bimodal assessment and comparison. Of the 17,881 genes analyzed, eight (*GSTM1, HLA-DRB1, ERAP2, HLA-DRB5, MAOA, ACTN3, NR4A2*, and *THNSL2*) were found to have bimodal expression replicated in the separate batch groups, while 24 other genes had evidence of bimodality in only one group. Some bimodally expressed genes had modest associations with pre-diabetic phenotypes, of note *ACTN3 *with insulin resistance. Most of the other bimodal genes have been reported to be involved with various other diseases and characteristics. Association of expression with *cis *genetic variation in a subset of 149 individuals found all but one of the confirmed bimodal genes and nearly half of all potential ones to be highly significant expression quantitative trait loci (eQTL). The rare prevalence of these bimodally expressed genes found after controlling for batch effects was much lower than the prevalence reported in other studies. Additional validation in data from separate muscle expression studies confirmed the low prevalence of bimodality we observed.

**Conclusions:**

We conclude that the prevalence of bimodal gene expression is quite rare in healthy muscle tissue (<0.2%), and is much lower than limited reports from other studies. The major cause of these clearly bimodal expression patterns in homogeneous tissue appears to be *cis*-polymorphisms, indicating that such bimodal genes are, for the most part, eQTL. The high frequency of disease associations reported with these genes gives hope that this unique feature may identify or actually be an underlying factor responsible for disease development.

## Background

RNA expression levels indicate the overall effect of a combination of genetic and environmental factors [1,2], which may result in distinct phenotypic differences, including disease susceptibility [3-5]. Attempts to unravel these genetic and environmental roles have met with only limited success for some complex diseases [6]. The emergence of new profiling technologies, including genome-wide expression arrays, has increased hope that these factors can soon be disentangled.

Our current study measured RNA expression in the largest genome-wide analysis of human skeletal muscle tissue of which we are aware [7-17]. In process of analyzing this data, we noticed that the expression levels for some genes showed a distinct bimodal distribution. This pattern was quite intriguing (a nearly ubiquitous assumption with genome-wide expression arrays is that expression-level data are normally or log-normally distributed), and led us to ascertain how often such patterns occurred, and whether such were associated with diabetes-related phenotypes. We also sought to determine the relative role that genetic factors had in the influence of these bimodal patterns.

Limited reports of bimodal expression patterns in other studies have also been made [18-20], particularly when heterogeneous tissues are being contrasted [21-26] such as between healthy and diseased tissue (and for which bimodal expression levels would be expected for perhaps many genes). However, in homogeneous tissue of apparently healthy individuals, such bimodality may be surprising, and hints at unrecognized heterogeneity which may involve precipitating factors of *future *disease development. Previous reports of bimodality [18,21-23,27] have suggested that 10-30% of transcripts show bimodal expression, yet with an apparent lack of replication of such findings [24]. As part of our analysis, we also observed that batch effects could potentially cause the artifactual detection of bimodality, inflating the prevalence estimate, but that after proper control, the prevalence of genuine bimodality in muscle tissue was in fact quite rare.

While this discrepancy in the estimated prevalence of bimodality is large and invites further investigation, the ability to distinguish genuine bimodals can lead to the more accurate evaluation of their biological role. Bimodality is an important biological phenomenon as it implies the existence of discrete populations (e.g., with and without disease) or discrete genetic or environmental influences on a trait. As such, bimodality has historically been sought in genetic research, yet few reproducible bimodal patterns have ever been observed in this field. One might, therefore, hope that genuine bimodally expressed genes will have significant genetic importance. Hence, it is most intriguing that many of the bimodal genes we identify herein have reported associations with a variety of human diseases and phenotypes. This elicits a multitude of hypotheses regarding the role of bimodal expression and human characteristics, including disease development. Our analysis also evaluates the role of bimodality in one such disease - type 2 diabetes, by assessing the association of the identified bimodal transcripts with pre-diabetic phenotypes in this population of non-diabetic Pima Indians who are at high risk for developing diabetes in their lifetime.

## Results

### Prevalence of bimodal expression

**Figure 1:**
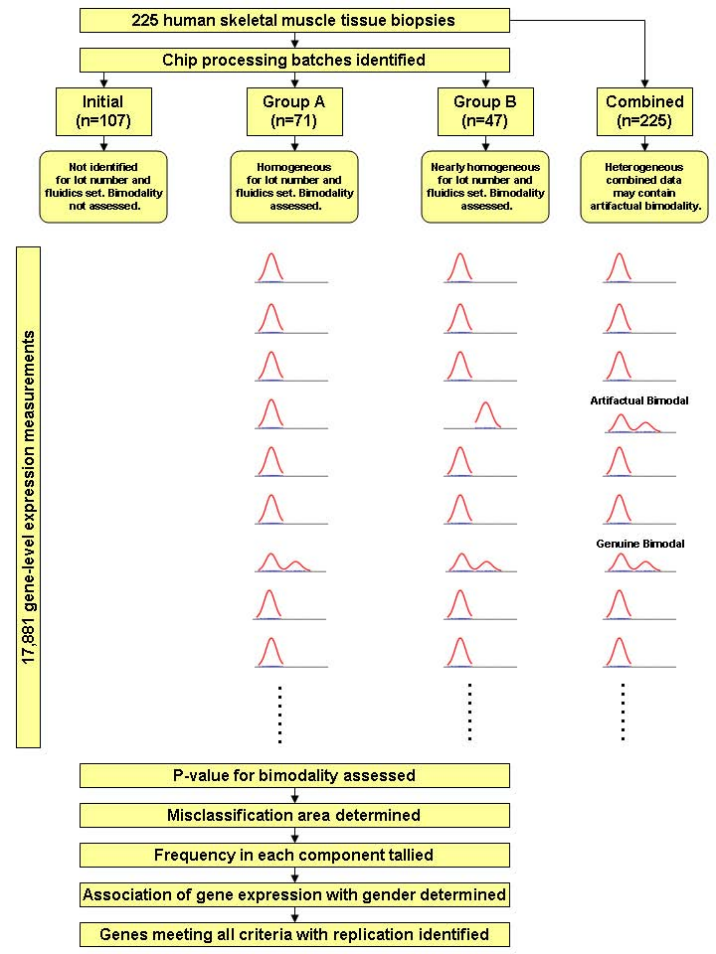
**Identification of genes with bimodal expression**. Muscle samples from 225 individuals were processed in groups that could be identified for distinct batch effects influencing the expression levels. Bimodality was assessed in groups of microarrays that were homogeneous for such bias. A variety of constraints were applied to ensure bimodal genes identified were genuine, including replication. Also illustrated is how bimodality may be artifactually (incorrectly) inferred due to heterogeneity of batch effects on expression levels.

The prevalence of bimodal expression was estimated from skeletal muscle biopsies taken from 225 non-diabetic, healthy Pima Indians. These tissue samples were scanned on Affymetrix Human Exon 1.0 ST microarray chips which provide on average 49 expression level measurements per gene. Subject characteristics are shown in Table 1. To assess bimodality of RNA expression, we fit unimodal and bimodal distributions to the gene-level expression data coming from each of the 17,881 core genes. We identified four clusters of chips by scan date which exhibited batch-type effects, two of which could be identified for lot numbers and fluidics station sets used in processing (Additional file 1). These batch effects were found to potentially cause spurious detection of bimodality, and hence the analyses were performed separately in two groups of identifiable chips of size 71 and 47. The full set of size 225 was also assessed for bimodality when such was observed in either of these independent groups (see Figure 1).

**Table 1:** Characteristics of 225 individuals with a baseline muscle biopsy.

Characteristic	Mean	Min	Max	N
**Age (years)**	29.6	18.1	49.7	225 (152 M, 73 F)

**Body Mass Index (kg/m**^**2**^**)**	33.4	19.1	55.0	225 (152 M, 73 F)

**% Body Fat**	31.8	9.4	47.3	223 (150 M, 73 F)

**Log**_**10 **_**M**_**low **_**(mg·kg EMBS**^**-1 **^**·min**^**-1**^**)**^**a**^	0.435	0.178	0.952	182 (123 M, 59 F)

**Acute Insulin Response (μU/ml)**^**b**^	288.9	89.9	864.3	79 (59 M, 20 F)

^a ^EMBS = estimated metabolic body size

^b ^in full heritage, normal glucose tolerant individuals with covariates

**Figure 2:**
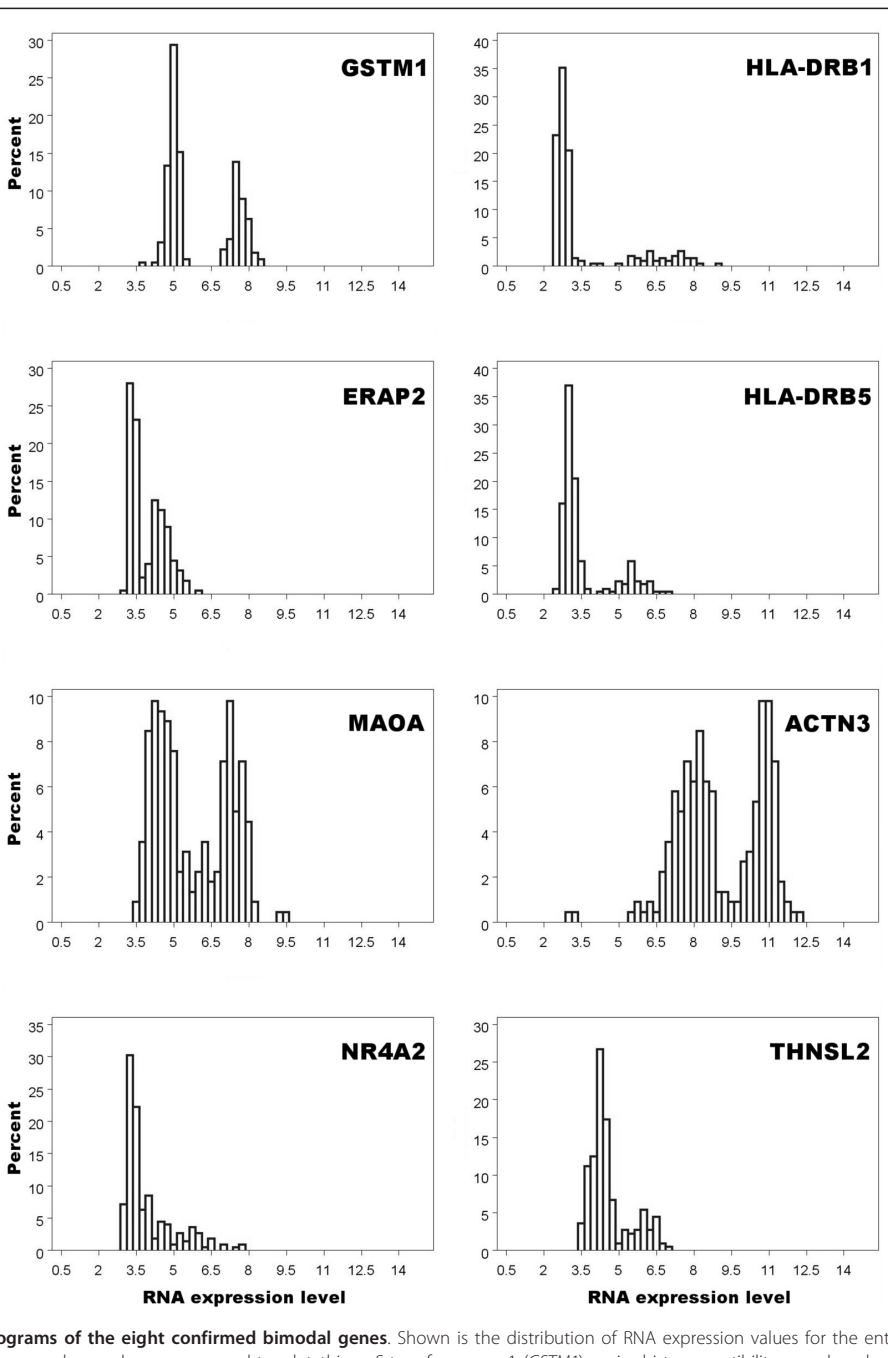
**Histograms of the eight confirmed bimodal genes**. Shown is the distribution of RNA expression values for the entire data set of n = 225. The 8 genes shown above correspond to: glutathione S-transferase mu 1 (*GSTM1*), major histocompatibility complex, class II, DR beta 1 (*HLA-DRB1*), endoplasmic reticulum aminopeptidase 2 (*ERAP2*), major histocompatibility complex, class II, DR beta 5 (*HLA-DRB5*), monoamine oxidase A (*MAOA*), actinin, alpha 3 (*ACTN3*), nuclear receptor subfamily 4, group A, member 2 (*NR4A2*), and threonine synthase-like 2 (*THNSL2*).

The total number of bimodally expressed genes estimated in each of the chip groups using different requirements for genuine bimodality is shown in Table 2. This analysis by group found only a small number of genes to be bimodally expressed with little overlap between the two components of the distribution, 19 in group A and 21 in group B, comprising a total of 32 unique genes (Table 3). Of these, 8 genes (*GSTM1, HLA-DRB1, ERAP2, HLA-DRB5, MAOA, ACTN3, NR4A2*, and *THNSL2*) were found to meet the stringent bimodal criterion in group A, group B, and the total study of 225 samples. Figure 2 shows the log_2 _expression level distributions for each of these confirmed bimodal genes. An additional 10 genes identified as bimodal had significant association with gender, of which 9 were located on the Y chromosome (Additional file 2).

**Table 2:** Number (prevalence) of bimodal genes as determined by compounding model selection criteria in the two different groups of microarrays.

(17,881 genes analyzed)		
Criteria for Bimodal Assessment	Group A	Group B
**p-val < 0.001**^**a**^	430 (2.40%)	40 (0.224%)

**+ misclassification area <0.1**^**b**^	408 (2.28%)	36 (0.201%)

**+ >10% points in each component**^**c**^	33 (0.185%)	33 (0.185%)

**+ gender p-val for heterogeneity >0.05**^**d**^	19 (0.106%)	21 (0.117%)

Columns represent the two groups of microarrays from which the bimodal genes were determined: Group A - 71 microarray chips all from the same lot, time span, and fluidics station; Group B - 47 microarray chips from two consecutive lots and from the same time span and fluidics station. The final row represents the total number of interesting bimodal genes for muscle expression in this population as estimated by the various chip groupings. A total of 17,881 genes were analyzed for potential bimodality.

^a ^p-value from a likelihood ratio test with 6 d.f.

^b ^calculated as proportion of misclassified area to unity

^c ^points in each component tallied as number to the left and right of the two components' intersection

^d ^from assessment in Group A

**Table 3:** Summary of 32 genes (not associated with gender) which met group criteria for bimodality.

Transcript number	Chromosome	Gene symbol	Group A p-value Bimodality N = 71	Group B p-value Bimodality N = 47	All chips p-value Bimodality N = 225	p-value Gender	Meets bimodal criterion in chip group(s)
**2350981**	**1**	***GSTM1***	**7.28E-19**	**4.79E-20**	**3.53E-72**	**0.808**	**A,B,All**

**4048265**	**6**	***HLA-DRB1***	**6.18E-16**	**5.61E-08**	**5.43E-44**	**0.822**	**A,B,All**

**2821347**	**5**	***ERAP2***	**9.33E-14**	**6.06E-06**	**1.87E-34**	**0.943**	**A,B,All**

**4048241**	**6**	***HLA-DRB5***	**4.87E-09**	**1.00E-08**	**1.41E-28**	**0.907**	**A,B,All**

**3975227**	**X**	***MAOA***	**2.83E-07**	**2.55E-05**	**1.42E-23**	**0.537**	**A,B,All**

**3336324**	**11**	***ACTN3***	**1.04E-06**	**7.09E-04**	**6.77E-20**	**0.275**	**A,B,All**

3618333	15	*MEIS2*	1.16E-06		1.35E-14	0.995	A,All

3103494	8	*TMEM70*	1.76E-06			0.382	A

2418570	1	*SLC44A5*	3.97E-06		4.17E-19	0.954	A,All

**2582124**	**2**	***NR4A2***	**1.09E-04**	**3.20E-04**	**6.00E-10**	**0.171**	**A,B,All**

3822074	19	*RAD23A*	2.26E-04			0.137	A

3682182	16	*ABCC6*	2.36E-04		1.86E-04	0.953	A,All

3590275	15	*CHAC1*	4.42E-04			0.724	A

3680953	16	*FLJ11151*	5.34E-04		9.34E-08	0.594	A,All

3393257	11	*BACE1*	5.67E-04			0.547	A

**2492783**	**2**	***THNSL2***	**6.03E-04**	**4.74E-04**	**5.27E-10**	**0.372**	**A,B,All**

3963990	22	*PKDREJ*	8.75E-04			0.403	A

3404660	12	*KLRD1*	8.85E-04			0.417	A

3737274	17	*KIAA1618*	9.33E-04			0.106	A

3824153	19	*C19orf62*		2.39E-06		0.367	B

3471427	12	*MYL2*		5.88E-06		0.144	B

3867223	19	*RPL18*		2.80E-05		0.841	B

2948887	6	*HLA-C*		5.15E-05	9.33E-04	0.929	B,All

3146433	8	*COX6C*		1.52E-04	1.90E-16	0.103	B,All

3402697	12	*COPS7A*		1.53E-04		0.773	B

3845782	19	*PLEKHJ1*		1.69E-04		0.090	B

2324616	1	*HSPC157*		1.87E-04	1.63E-05	0.062	B,All

2431031	1	*HMGCS2*		2.32E-04	3.59E-04	0.202	B,All

3373392	11	*OR8H1*		4.63E-04		0.581	B

2344888	1	*CYR61*		5.60E-04		0.611	B

3851055	19	*ELOF1*		5.98E-04		0.699	B

2383726	1	*ARF1*		9.45E-04		0.078	B

P-value for bimodality is shown for each group of microarray chips when all criteria for bimodality were met. Blank cells indicate that at least one bimodal criterion for the given gene and group was not met. Genes identified as bimodal in all groups are shown in bold.

As bimodal expression may reflect either two distinct levels of transcript abundance or a single transcript abundance level with the lower mode reflecting no expression, we compared the median levels of the lower mode of the confirmed bimodal genes to the median expression levels of all other genes (n = 17,783). The levels of the lower mode were indicative of background (no gene expression) for half of these confirmed bimodals - having median expression levels at the 25^th ^or lower percentile of all genes, while four of them (*THNSL2*, *MAOA*, *GSTM1*, and *ACTN3*) appeared to have RNA expression at two distinct abundance levels, each with lower modes corresponding to levels near or above the median of the high mode of *ERAP2 *(55^th ^percentile).

**Figure 3:**
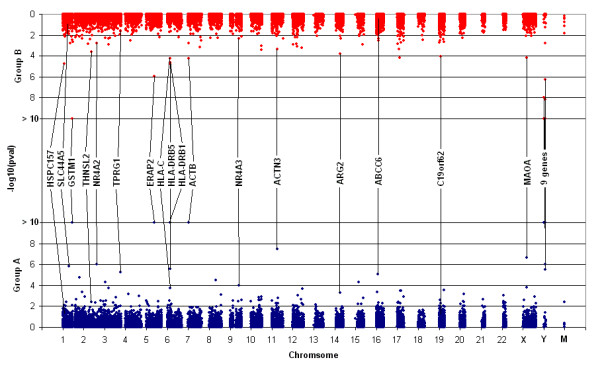
**Two-way Manhattan plot of significance for bimodality in both of the independent groups**. Shown are the -log_10 _p-values for bimodality for each of the 17,881 genes from trimmed Group A (blue circles, n = 65) and from trimmed Group B (red circles, n = 43). Genes with a combined Fisher's FDR < 0.05 are labeled (see Additional files 3 and 4).

Many genes initially classified as bimodal in group A had very few data points (often 1 or 2) lying to the left or right of the intersection of the two component normal distributions. As discussed in Methods, genuine bimodality should not reflect the potential influence of a small number of outliers. For a robust reassessment of bimodality, we deleted the lowest 5% and highest 5% of expression values by chip group for each gene. Bimodality was again assessed in these trimmed data sets (n = 65 and n = 43), with similar bimodal prevalence estimates found to the estimate which required a minimum of 10% of data points to be in each component (Additional files 3 and 4). A combined Fisher analysis of these trimmed data sets found the same set of 8 confirmed bimodals plus an additional 6 genes (*HLA-C, SLC44A5, NR4A3, HSPC157, ABCC6*, and *C19orf62*) to have a false discovery rate (FDR) < 0.05. The p-values for bimodality of all 17,881 genes in both independent batch groups are plotted in Figure 3.

### Diabetes related phenotypes and mode of expression

We assessed the association of the mode of expression with pre-diabetic traits (body mass index/percent body fat, insulin resistance, and insulin secretion), in the eight confirmed bimodal genes (Table 4). We found insulin sensitivity as measured by a hyperinsulinemic, euglycemic clamp to be higher in those in the low mode of *ACTN3 *expression (p = 0.0076) and percent body fat to be higher in those in the high mode of *MAOA *expression (p = 0.018). Body mass index and acute insulin response to an intravenous glucose tolerance test were not found to be associated with any of the bimodal genes in this analysis.

**Table 4:** Differences (p-value) in diabetes related traits in the 8 confirmed bimodal genes for those in the high compared with those in the low RNA expression mode.

Gene	**Δ BMI**^**a**^	**Δ %fat**^**a**^	**Δ log**_**10**_**(M**_**low**_**)**^**b**^	**Δ AIR**^**c**^
***GSTM1***	-0.408 (p = 0.67)	-0.88 (p = 0.28)	0.015 (p = 0.46)	-1.73 (p = 0.96)

***HLA-DRB1***	2.04 (p = 0.09)	1.15 (p = 0.32)	-0.0033 (p = 0.89)	31.6 (p = 0.49)

***ERAP2***	-0.122 (p = 0.9)	0.472 (p = 0.58)	-0.0033 (p = 0.86)	54.4 (p = 0.11)

***HLA-DRB5***	2.19 (p = 0.072)	1.22 (p = 0.29)	-0.0095 (p = 0.69)	31.6 (p = 0.49)

***MAOA***	0.949 (p = 0.29)	**1.78 (p = 0.018)**	-0.0001 (p = 1.0)	-44.1 (p = 0.17)

***ACTN3***	0.71 (p = 0.46)	0.284 (p = 0.72)	**-0.050 (p = 0.0076)**	53.5 (p = 0.14)

***NR4A2***	-0.576 (p = 0.57)	-0.836 (p = 0.36)	-0.010 (p = 0.63)	-2.05 (p = 0.96)

***THNSL2***	0.695 (p = 0.47)	0.671 (p = 0.48)	0.0062 (p = 0.81)	2.23 (p = 0.97)

The number of individuals measured for each phenotype varied (n).

^a ^adjusted for age, gender, sibship, and heritage; BMI = body mass index (n = 225), %fat = percent body fat (n = 223)

^b ^adjusted for age, gender, sibship, heritage, and % body fat; M_low _= insulin sensitivity (n = 182)

^c ^adjusted for age, gender, sibship, heritage, % body fat, and M_low_; AIR = acute insulin response (n = 79 normal glucose tolerant individuals with full heritage and covariates)

### Association of bimodal expression and *cis *determinants

To investigate the potential cause of such bimodal expression, a subset of 149 individuals who were also genotyped (Affymetrix Genome-wide Human SNP Array 6.0) were analyzed for association with muscle expression levels. *Cis-*acting SNPs (defined as lying within a 200 kb region to either side of the gene location) were sought in this population. Nearly all of the confirmed bimodally expressed genes and half of the entire bimodal set had highly significant (unadjusted p < 10^-8^) associations between muscle tissue expression and SNP genotype (Table 5 and Additional file 5), and were hence expression quantitative trait loci (eQTL). Using the intersection of the two underlying normal distributions as the threshold for assigning individuals to either component of the bimodal distribution, the coefficient of agreement between expression component and SNP genotype was high (κ > 0.80) for the majority of the confirmed bimodal genes (Table 5). Still, some genes had little or less than expected agreement for the density of coverage of the SNP chip. For example, *NR4A2 *had no agreement at all (κ = 0.0). We further investigated this gene for any *trans*-determinant cause across the entire genome, but no SNP approached a genome wide significance level. We also noted that while *GSTM1 *had the clearest separation of expression modes, it had the lowest kappa coefficient of the seven *cis*-associated bimodals, possibly indicating influences other than nearby SNPs on the expression levels, such as environmental exposure or more complex genetic models.

**Table 5:** Associations of 32 bimodal genes with *cis*-determining SNPs and reported CNV.

Bimodality confirmed in all data sets	Gene symbol	SNP association with expression **p-val**^**a**^	Kappa **coeff**.	# of SNPs within 200 KB	**Reported CNV**^**b**^
**Yes**	***GSTM1***	**1.20E-11**	**0.65**	**36**	**Yes**

**Yes**	***HLA-DRB1***	**<1E-16**	**0.83**	**119**	**Yes**

**Yes**	***ERAP2***	**<1E-16**	**0.85**	**94**	**No**

**Yes**	***HLA-DRB5***	**2.12E-14**	**0.86**	**104**	**Yes**

**Yes**	***MAOA***	**<1E-16**	**0.80**	**69**	**No**

**Yes**	***ACTN3***	**<1E-16**	**0.90**	**9**	**No**

	*MEIS2*	0.04	0.17	86	No

	***TMEM70***	**<1E-16**	**0.39**	**76**	**No**

	***SLC44A5***	**<1E-16**	**0.74**	**112**	**Yes**

Yes	*NR4A2*	0.26	0.00	36	No

	*RAD23A*	0.22	0.09	13	No

	***ABCC6***	**2.88E-10**	**0.36**	**59**	**Yes**

	*CHAC1*	0.035	0.12	12	No

	***FLJ11151***	**8.62E-7**	**0.43**	**181**	**No**

	*BACE1*	0.0037	0.17	79	No

**Yes**	***THNSL2***	**<1E-16**	**0.95**	**74**	**No**

	*PKDREJ*	0.023	0.02	24	Yes

	*KLRD1*	0.084	0.01	61	No

	*KIAA1618*	0.088	0.09	63	Yes

	*C19orf62*	0.022	0.01	41	Yes

	*MYL2*	0.061	0.00	52	No

	*RPL18*	0.17	0.10	19	Yes

	***HLA-C***	**<1E-16**	**0.51**	**153**	**Yes**

	*COX6C*	0.000097	0.15	36	No

	*COPS7A*	0.048	0.04	47	No

	*PLEKHJ1*	0.048	0.14	22	Yes

	***HSPC157***	**<1E-16**	**0.84**	**64**	**Yes**

	*HMGCS2*	0.0026	0.25	76	Yes

	*OR8H1*	0.049	0.23	49	No

	*CYR61*	0.012	0.11	100	No

	*ELOF1*	0.019	0.04	32	No

	*ARF1*	0.065	0.12	55	Yes

^a ^Highest SNP association shown. All other significant SNPs are listed in Additional file 5. Apparent *cis*-acting eQTL are identified in bold.

^b ^Copy number variation (CNV) as identified in the UCSC Genome Browser.

### Prevalence of copy number variation in bimodal genes

As polymorphic deletions may greatly affect expression levels, we sought to determine the frequency with which such copy number variation (CNV) may be responsible for producing the bimodal expression patterns. The genes listed in Table 3 were assessed from the UCSC Genome Browser for structural variation (gain, loss, or both) that involved segments of DNA larger than 1 kb. Of the 8 confirmed bimodally expressed genes, copy number variation is known to be present in 3, while 18 of the total 32 possible bimodal genes have currently identified copy number variation. As a comparison, we also assessed a random sample of 32 genes with normally distributed gene expression, and found the same number of genes - eighteen, with reported CNV in this database, indicating no difference in the prevalence of CNV in bimodal as compared to unimodally expressed genes. Hence while CNV may well cause bimodal expression for some genes (most notably *GSTM1*), for the majority of confirmed bimodals, known CNV is likely not the explanation for such a distribution.

### Validation in other populations

We further assessed bimodal RNA expression in two publically available data sets containing expression data from healthy muscle biopsies (GSE13070 [14]; n = 59 and GSE5086 [15]; n = 62). We found the prevalence of bimodal RNA expression in these populations to be 0.72% and 1.4% respectively. These estimates were similar to the prevalence we observed, providing further validation that bimodal RNA expression in healthy muscle tissue is a rare event. We also re-assessed bimodal RNA expression in healthy lymphoblastoid cells (GSE1485 [28]; n = 193) which had previously been estimated to have high bimodal prevalence [18]. We found the prevalence of bimodality in the restricted genes of that analysis to be only 2.9% by our criteria, the difference largely reflecting a previously used statistical threshold which is associated with a much higher false discovery rate than that used in other bimodal expression studies. Of great interest, we found that many of the bimodal genes of our analysis were also highly significant for bimodality in these other three populations (Table 6). These additional validations are most compelling as clear bimodality was also rare in these other populations, with p < 10^-8 ^of chance concordance of the bimodal gene sets in each of these populations with the Pima bimodals (Additional file 6). We also note that while the most clearly bimodal gene replicating in our analysis (*GSTM1*) did not show strict bimodality in these other populations, related genes were highly bimodal in these other data sets (e.g. *GSTM2 *in GSE1485 and *GSTT1 *in GSE5086, data not shown).

**Table 6:** Further validation of genes having bimodal expression (FDR < 0.05) in Pima Indians with bimodal expression of those genes in other populations.

Gene symbol	**Chr**.	Pima Bimodality **p-value**^**a**^	GSE1485 Bimodality **p-value**^**b**^	GSE13070 Bimodality **p-value**^**c**^	GSE5086 Bimodality **p-value**^**d**^	GSE1485 ID	GSE13070 ID	GSE5086 ID
Genes with no Association of Expression and Gender								

*GSTM1*	1	3.54E-44	0.042	0.532	0.405	204550_x_at	215333_x_at	215333_x_at

***ERAP2***	5	8.76E-18	**2.19E-26**	0.038	0.00768	219759_at	227462_at	227462_at

***HLA-DRB1***	6	8.33E-15	**1.74E-13**	0.859	0.515	204670_x_at	208306_x_at	208306_x_at

***ACTN3***	11	3.41E-10	-	**3.34E-06**	**9.09E-08**		206891_at	206891_at

***MAOA***	X	3.97E-10	-	**9.55E-06**	**5.48E-04**		204388_s_at	204389_at

*HLA-DRB5*	6	1.20E-09	-	-	-			

***NR4A2***	2	3.21E-08	-	**1.37E-04**	0.468		216248_s_at	204622_x_at

***HLA-C***	6	2.02E-07	**1.84E-07**	0.471	0.607	216526_x_at	216526_x_at	216526_x_at

*SLC44A5*	1	1.76E-06	-	0.324	0.043		235763_at	235763_at

*NR4A3*	9	6.63E-06	-	0.690	0.347		207978_s_at	207978_s_at

*HSPC157*	1	1.13E-05	-	0.987	0.204		219865_at	219865_at

*THNSL2*	2	1.42E-05	-	0.022	0.315		219044_at	239949_at

*ABCC6*	16	3.73E-05	-	0.721	0.045		208480_s_at	208480_s_at

*C19orf62*	19	5.60E-05	-	0.230	0.094		221711_s_at	221711_s_at

Genes with Association of Expression and Gender								

***DDX3Y***	Y	7.86E-54	**4.27E-86**	**1.31E-16**	**2.30E-18**	205000_at	205000_at	205000_at

***EIF1AY***	Y	3.91E-49	**1.36E-75**	**1.02E-28**	**2.87E-23**	204409_s_at	204409_s_at	204409_s_at

***RPS4Y1***	Y	7.50E-45	**1.20E-138**	**1.22E-34**	**7.20E-42**	201909_at	201909_at	201909_at

***UTY***	Y	1.15E-42	**8.33E-05**	0.00269	**1.39E-14**	211149_at	211149_at	211149_at

***USP9Y***	Y	1.46E-32	**1.68E-22**	**2.81E-09**	**5.08E-16**	206624_at	228492_at	228492_at

***ZFY***	Y	6.01E-22	-	**3.86E-04**	**1.89E-12**		230760_at	230760_at

*CYorf15B*	Y	9.51E-21	-	0.0324	0.00508		214131_at	223646_s_at

***ACTB***	7	1.94E-14	-	0.111	**3.75E-04**		AFFX-HSAC07/X0	AFFX-HSAC07/X0

*TTTY10*	Y	1.48E-11	-	0.194	0.798		224293_at	224293_at

***CD24***	Y	9.99E-08	0.998	**1.18E-04**	0.0101	208650_s_at	216379_x_at	266_s_at

***ARG2***	14	1.23E-06	-	**1.45E-04**	0.910		203946_s_at	203945_at

*TPRG1*	3	2.10E-06	-	0.0353	0.961		229764_at	229764_at

^a ^Present study in skeletal muscle tissue of non-diabetic Pima Indians. P-value from Fisher's meta-analysis of trimmed Group A (n = 65) and trimmed Group B (n = 43) for bimodality of RNA expression; shown are all genes having an FDR < 0.05. See additional files 3 and 4.

^b ^Bimodality of RNA expression assessed in lymphoblastoid cells from CEPH Utah residents (n = 193). Not all genes were represented on the chip or had excessive intra-individual variation (-). ID shown is affy_id. P-values < 0.001 from a likelihood ratio test with 6 d.f. shown in bold. GSE1485 expression data provided to GEO as part of unrelated study [28].

^c ^Bimodality of RNA expression assessed in abdominal muscle from patients undergoing gastrointestinal surgery (n = 59). Not all genes were represented on the chip (-). ID shown is affy_id. P-values < 0.001 from a likelihood ratio test with 6 d.f. shown in bold. GSE13070 expression data provided to GEO as part of unrelated study [14].

^d ^Bimodality of RNA expression assessed in skeletal muscle from subjects with a broad range of insulin sensitivity/resistance (n = 62). Not all genes were represented on the chip (-), ID shown is affy_id. P-values < 0.001 from a likelihood ratio test with 6 d.f. shown in bold. GSE5086 expression data provided to GEO as part of unrelated study [15].

## Discussion

Our detection of bimodal gene expression in skeletal muscle tissue reveals an apparently rare (<0.2%), yet potentially new predictor of human disease and phenotypes. Of the eight confirmed bimodal genes, we found that seven were expression quantitative trait loci, as they were strongly to completely associated with possibly causal SNPs occurring within a 200-kb *cis *region of the gene. This finding suggests that the majority of bimodal genes which may be found in healthy human tissue may be the result of polymorphisms within such gene regulatory regions. In the remaining potential bimodal genes, highly significant SNP associations were less common (possibly reflecting misclassification of bimodal genes), though still more frequent than occurs genome-wide. Hence in healthy individuals, eQTL are prime (yet not exclusive) locations for finding bimodally expressed genes. We also found that known copy number variation - while a potential explanation for some bimodal expression, was not found in higher abundance than across the genome, and is hence likely not the main source of such distributions.

The current study also assessed the relationship of RNA expression mode with known predictors of diabetes. We observed a likely association of insulin sensitivity with mode of *ACTN3 *expression, with 42.2% of the individuals falling in the higher expressed mode which was associated with insulin resistance. While not meeting strict correction for multiple testing, this gene has previously been associated with slow- and fast-twitch muscle fiber, with correlation to athletic performance [29]. The bimodal expression of *ACTN3 *could reflect a bimodal distribution in the proportion of type 2 fibers present in the subjects, though we are not able to assess this directly. As insulin resistance is a known predictor of diabetes [30], the role of *ACTN3 *expression deserves further investigation.

Many of the other bimodal genes identified have previously been reported to be associated with other diseases and characteristics ranging from cancer to arthritis [31-39], some of these also reporting marked dichotomies in expression levels [38,39]. Perhaps the most intriguing disease-related bimodal genes are the *HLA *loci, as the *HLA *system is known not only to convey risk for a variety of immune-related diseases, but contains the main genetic risk factors for type 1 diabetes [32]. That three of the *HLA *genes (*DRB1, DRB5*, and *C*) were found to have bimodal distributions - as well as *ERAP2 *which plays a final role in producing *HLA *peptides [34], is of great interest, and we are currently further investigating the role of these genes in relation to diabetes and various metabolic phenotypes in the Pima population.

The prevalence of bimodal gene expression we have observed in a healthy single tissue is approximately 100-times lower than what has been reported in mixed-tissue studies [21,22]. Since these studies assessed distinct expression differences across heterogeneous tissues, a larger number of bimodally expressed genes would be expected. Our consistently low estimate gives confidence that at least in healthy muscle tissue, unambiguous bimodal expression is not common - providing a new reference prevalence for bimodality in healthy tissue to which studies of bimodality in diseased and mixed tissues may be compared.

Our estimate also differs greatly from the only other prevalence reported for bimodal expression in a population comprised solely of healthy persons - 28% in lymphoblastoid cells [18,28]. As the threshold for assessing bimodality in that study was much more liberal, we re-analyzed the data from this population and found only 2.9% of genes of the limited gene set previously analyzed to show bimodality consistent with the thresholds used in our analysis (see Methods). We also assessed two other publically available data sets containing muscle biopsies, finding estimates of clear bimodality in these populations to be 0.72% and 1.4%, and again with <0.2% prevalence with replication of bimodality across these two data sets (data not shown). These validation sets used chips which had on average 1 or 2 probes per gene, whereas our present study is the first to assess bimodality in expression data from exon arrays which contained on average 49 probes per gene. Hence, the bimodality we observed represents a strong replicated signal which could be missed by a single probe, yet could also miss uniquely bimodal probes when averaged at the gene level. The additional validation of our confirmed bimodal genes in these other populations with low bimodal prevalence (Table 6) provides even more convincing evidence of their authenticity.

We highlight two technical issues for future studies to be aware of: use of a model that allows for unequal variances in the bimodal distribution and the determining of batch effects. We observed that a model that allows for unequal variances was clearly more appropriate than a constant variance model for approximately 20% of the genuine bimodal genes identified in our data, and hence the failure to allow for such as well as differences in data scaling may contribute to some variability in prevalence estimates. Even more important is the potential of batch effects to cause artifactual bimodal expression. Such confounding can occur when any set of experimental factors is not uniform - causing the expression levels to be uniformly over- or under-estimated for a given transcript for a portion of the samples. We noted that these batch effects were associated more frequently with genes of higher transcript abundance levels. We also identified that chips which had distinct lot number differences and which had been processed on distinctly different fluidics station sets gave rise to such artifactual bimodality, though it was not possible to determine which of these factors had a more primary role in this study. However, because this grouped source of heterogeneity was identified for most arrays, bimodality could be analyzed in homogeneous groups, eliminating not only the unwanted bias, but also providing distinct replication sets in which tests for bimodality could be confirmed.

## Conclusions

In conclusion, the prevalence of bimodal RNA expression in homogeneous tissue is quite low, and hence has been unrecognized in most expression analyses. The few prior genome-wide studies which have reported bimodality may have over-estimated its prevalence, possibly due to the presence of batch effects and/or overly lenient statistical thresholds. Differences in cell type, microarrays, and populations may also be responsible for some of the large difference in estimated prevalence. An additional novel insight from this analysis is that the majority of genes with bimodal RNA expression apparently exhibited this pattern due to *cis*-polymorphisms, being expression quantitative trait loci. Future investigations of bimodality in homogeneous tissues from healthy persons will be of great interest in assessing similarities and differences across other tissues and populations, as well as their association with other human characteristics and diseases. In the Pimas, the association of a bimodal gene with insulin resistance and the presence of bimodality in genes from the HLA locus which carries the greatest risk for type 1 diabetes underscore the need to further investigate and understand this interesting phenomenon.

## Methods

### Subjects

All subjects were part of our study of the etiology of type 2 diabetes among Pima Indians living in Arizona. Volunteers providing muscle biopsies were non-diabetic, healthy, and were not taking any medications. Subjects were assessed for diabetes-free status via a fasting blood draw as well as a 75 gram 2 hour oral glucose tolerance test (OGTT) to confirm that the subject was indeed non-diabetic (fasting glucose <126 mg/dl and 2 hour glucose <200 mg/dl). All subjects provided informed consent (which places some restrictions on access to data and intended research purposes) and all studies were approved by both the institutional review board of the National Institute of Diabetes and Digestive and Kidney Diseases and the council of the Gila River Indian Community.

### Tissue Samples

Most muscle biopsies were performed within one week's time of the diabetes assessment (all biopsies were performed within 30 days). After a 12 hour overnight fast and monitored physical inactivity, percutaneous needle biopsies were carried out on the vastus lateralis muscle under local anesthesia with 1% lidocaine. The biopsy specimens were snap frozen and stored at -20 to -70°C. Variation in storage time ranged up to 16 years, however no effect of the amount of time frozen on RNA quality was found.

### RNA Isolation

Muscle tissue samples were homogenized using a PT 1300D (Brinkmann, Westbury, NY, USA) homogenizer with 12 mm rotor/stator head in 2.0 ml of cold TRIzol reagent (Invitrogen/Life Technologies, Carlsbad, CA, USA). The samples were then heated to 65°C and 0.4 ml chloroform (Sigma, St. Louis, MO, USA) was added. 5 μL of 4 ng/μL Carrier RNA was added to each sample to aid precipitation with ETOH and further purified using RNeasy Micro Kit (Qiagen, Valencia, CA, USA). 1 μg Total RNA Labeling Protocol starts with a ribosomal RNA (rRNA) reduction procedure where the 28S and 18S rRNA portion is significantly reduced from the total RNA sample minimizing the background and increasing the array detection sensitivity and specificity. All RNA was isolated by a single technician.

### Expression Measurements

The rRNA was prepared and the cDNA synthesized for the Human Exon 1.0 ST Array microarray chips (Affymetrix, Santa Clara, CA, USA) using the GeneChip Whole Transcript Sense Target Labeling Assay kits (Affymetrix). Chip washing and staining was performed in two distinct sets of fluidics stations (GeneChip Fluidics Station 450, Affymetrix) and scanned on as many as five different scanners (GeneChip Scanner 3000 7G, Affymetrix). Two technicians performed the scanning measurements in discrete sets of dates.

Quality control metrics and technician insight were used to determine the need for re-scans. When re-scanning did not resolve the problem, such chips were discarded and cDNA hybridization mix placed on completely new chips (this occurred in 9.8% of the samples). A total of 225 baseline samples which passed such quality control metrics resulted in expression results available for analysis. Only transcript clusters identified as "core" (genes which have been identified with a high level of confidence) were analyzed, comprising a total of 17,881 genes.

### Normalization Techniques

To account for varying intensity levels and other variability between different scans, we normalized the 225 chips using the Robust Multichip Average method with quantile normalization of the log_2 _intensities with prior GC correction (GC-RMA [40,41]), using the mean of all probes associated with the given gene. Visual inspection of chip intensities found some chips with limited smears or mars (a common occurrence in such experiments), however, the bimodal methods employed are likely to be robust to such occasional random technical events, and hence no chips or regions of chips were excluded.

### Batch Effect

In analyzing the 225 muscle files, we discovered a batch effect which was associated with distinctly different expression values by clusters of sample processing dates for some genes (Additional file 1). Investigating this batch effect, we determined that the final two clusters consisted of microarrays with distinctly different chip lot numbers and which had been processed on different fluidics stations. Initially (i.e. before discovering that a batch effect was present), use of all available chips led to the false detection of many bimodal genes - as combined expression values of normally distributed genes from different batches produced an apparent overall bimodal distribution. Hence, we conducted our analyses on the expression data from each batch separately - providing not only an independent confirmation set, but also an analysis free from this bias. While common linear adjustments may remove batch effects for normally distributed data, due to natural unequal proportions of data lying in each component of bimodal data, similar adjustments could result in bimodal data from two batches having 4 modes. Hence, we restrained our analysis to unadjusted data in separate batches.

The remaining 107 microarray chips could not be identified for lot number or for the fluidics station set they were processed on. Hence, we did not explore bimodality in this data separately, but constrained our initial analysis to the two identifiable clusters that had also been processed by a single technician (a cluster of 47 chips - group A, and another cluster of 71 chips - group B). While differences between clusters were observed for some genes, other genes appeared to be free from this batch effect. For these unaffected genes, power to detect associations with true bimodality would be greater in the total data set (225 chips) due to increased sample size. Hence, we first confirmed bimodality in the separate clusters before using the entire data set for further confirmation and association analyses.

### Unimodal and Bimodal Distribution Fitting

For each gene, we had 47, 71, or 225 data points corresponding to the expression levels coming from each participant in the two batch groups or in the entire study. To avoid skewness with a unimodal distribution resulting in an artifactual inference of bimodality, the log_2 _expression data for each gene were transformed by the Box-Cox method to reduce skewness [42]. The Box-Cox parameter was systematically varied in increments of 0.01 over the range of -4 to 4 to select the most appropriate value for each gene. Subsequently, maximum likelihood methods were used to estimate the parameters of a unimodal Gaussian distribution and of a bimodal Gaussian distribution (allowing for unequal variances of the two modes) that were most consistent with the transformed data. The unimodal density distribution is described by two parameters, the mean (μ) and standard deviation (σ), while the bimodal involves the means and standard deviations in each component (μ_1_, σ_1_, μ_2_, σ_2_) and the proportion of area in one of the components (*e.g.*, p_1_). Such methods for model fitting have been used previously [43,44]. A potential difficulty with maximum likelihood techniques is that it is difficult to be certain that the model has converged at the global, rather than a local, maximum of the likelihood. To ensure such convergence, parameters were estimated using a novel automatic parameter estimation technique. Essentially this method calculates the likelihood at various plausible values of the parameters chosen systematically (200,000 estimations per gene for the present study); the optimal values are then taken as initial estimates for a final maximum likelihood estimation using the Newton-Raphson algorithm. The result of this method was to greatly increase the likelihood of converging to the global maximum, with a coinciding increase in computational time that was not prohibitively long for the bimodal model (time was less than 1 minute per gene).

### Model Selection

There is no unequivocal selection test for bimodal versus unimodal distributions, despite the fact that the models are nested [22,24,45]. Hence, a number of different criterion have been proposed, which we adopted. These include using a p-value threshold of 0.001 from a chi-square distribution with six degrees of freedom (as opposed to three in order to provide a more stringent cutoff) on the minus 2 log likelihood difference from the models, as well as a simultaneous requirement of misclassification area <0.1 based on the best estimated bimodal distribution [21,22,45]. This area is calculated as the minimum area under either curve to the right or left of the intersection of the two separate modes. The number of intersections can be 0, 1, or 2. When 0, the mean values were used as the dichotomous intersection estimates. When multiple, both intersections were used, with the intersection providing the minimal misclassification selected.

Additionally, we introduced a new requirement that the minimum number of data points to the left and right of the selected intersection point be at least 10%. This prevented bimodal model selection when a single or small number of outliers would otherwise have caused bimodal selection. Such a requirement is necessary to eliminate potentially large numbers of bimodal misclassifications for genes in which the best fit distribution's misclassification area is not significant, yet the bimodal fit is clearly the result of a single or small number of outlier points. As a consequence, this additional criterion will permit only the detection of bimodal genes that are dichotomously expressed in a non-trivial percentage of individuals, yet with greater confidence that these genes are not mis-assessed due simply to outlier points.

We confirmed the need for such a strategy in a separate analysis in which the lowest 5% and highest 5% of expression values were dropped. Such a strategy would prevent misclassification of bimodality due to a small number of outliers, though it would reduce the power to detect such in clean data. We noted this method to give very similar end-result prevalence estimates.

As some genes are expressed differently by gender, we fit a separate linear model on GC-RMA normalized expression levels to assess this association, using the resulting two-sided p-value as an additional tool to determine which genes exhibit bimodal expression due to different expression levels in men and women. If this p-value was ≥ 0.05, it was assumed that the bimodality was not due to gender.

### SNP Associations

A total of 149 individuals with expression data were also genotyped with Affymetrix Genome-wide Human SNP Array 6.0 chips. Potential *cis*-acting SNPs (expression quantitative trait loci, [46]) were identified as falling within 200 kb regions upstream or downstream of the start and stop locations of each bimodal transcript. A linear model was used to assess the association with adjustment for age, gender, and heritage with sibship included as a random effect to account for relations. A total of 1,221,921 SNP-expression associations were tested, from which a conservative Bonferroni estimate of significance would be 4.09 × 10^-8^. Expression levels were rank transformed with subsequent back-transformation to a normal distribution prior to fitting the linear models.

### Physiological Measurements

Assessment of pre-diabetic traits was made as part of the research protocol corresponding to the investigation nearest to the muscle biopsy, usually performed on the same day, and never greater than 30 days from the biopsy date. Insulin action in vivo was assessed using the hyperinsulinemic, euglycemic clamp as described in [30]. Acute insulin secretion was assessed during an intravenous glucose tolerance test with analysis restricted to those with normal glucose tolerance (OGTT <140 mg/dl) and who were full heritage Pima [47]. Percent body fat was performed via underwater weighing and total body dual energy X-ray absorptiometry [48].

### Additional Validation

We assessed bimodality in three additional data sets accessed from the Gene Expression Omnibus (GEO, http://www.ncbi.nlm.nih.gov/geo/). We re-analyzed bimodality in the expression of lymphoblastoid cells from 193 CEPH Utah residents (GSE1485, [18,28]) and analyzed for the first time bimodality in 59 normal abdomen muscle biopsies obtained during gastrointestinal surgeries (GSE13070, [14]) and 62 skeletal muscle biopsies in a population with broad range of insulin sensitivity/resistance (GSE5086, [15]). The expression data files analyzed came from individuals prior to treatment when such occurred in the study designs and for which data were available. Criteria for bimodality were synonymous with those of the present study. When multiple probes for a gene were present in these data sets, the probe giving the best evidence of bimodality was used. Only 3,554 genes from GSE1485 which had less intra-individual variation (assessed from replicates) than inter-individual variation were analyzed, as was previously done [18,28]. This would infer that our re-computed prevalence of 2.9% bimodality in this gene subset overestimates the total genome-wide prevalence of bimodal expression in that data. A total of 41,789 probes were analyzed in the GSE13070 and GSE5086 data sets. Probability of concordance of bimodal genes between the data sets was assessed via Fisher's exact test [49].

### Additional Analyses

Fisher's method was used to combine p-values from independent chip groups. False Discovery Rates [50] were assessed in the 12,470 genes that were not associated with gender (p ≥ 0.05) and separately in the 5,411 genes that were associated with gender (p < 0.05). All other p-values listed are not adjusted for multiple comparisons. General linear models were used to determine association of traits with expression mode after adjustment for appropriate covariates with sibships treated as repeated measures. Coefficients of agreement between genotype and mode of expression were calculated as the maximum kappa-Cohen statistic of the four possible groupings of dominant models and mode pairings. Copy number variation was assessed from the UCSC Genome Browser (2006 Assembly) as structural variation (gain, loss, or both) that involved segments of DNA larger than 1 kb.

### Computer Software

Gene scans were performed using Affymetrix GCOS software and Affymetrix Expression Console. GC-RMA normalization was performed using Partek Software Version 6.4 (6.09.0422). SAS Version 9.1 was used for Box-Cox transformations, Newton-Raphson maximum likelihood convergence, pre-diabetic phenotype associations, Fisher's exact test, as well as for all SNP association analyses. Code was also written in Microsoft Visual Studios C++ for data manipulation and maximum likelihood with automatic parameter estimation distribution fitting of unimodal and bimodal models.

## Authors' contributions

Data were acquired by CCM, CB, JK, LB, and VO; data were analyzed by CCM; interpretation of the data was made by CCM, RLH, and CB; CB and LJB provided funding; CCM conceived the bimodal study, designed the figures, and wrote the paper, with advice given from CB and RLH. All authors read and contributed to the manuscript.

## Supplementary Material

Additional file 1 **Figure illustrating how dichotomous gene level RNA expression can be artifact of batch effect**. Click here for file

Additional file 2 **Summary of 16 genes (associated with gender) which met all criteria for bimodality in either group A or group B**. Click here for file

Additional file 3 **Summary of bimodal genes found on trimmed bimodal data sets of non-gender associated genes**. Listed are 28 genes which had a false discovery rate on the Fisher combined p-value from the two trimmed data sets <1.0. Fourteen of these have an FDR < 0.05. Click here for file

Additional file 4 **Summary of bimodal genes found on trimmed bimodal data sets of gender associated (p < 0.05) genes**. Listed are 30 genes which had a false discovery rate on the Fisher combined p-value from the two trimmed data sets <1.0. Twelve of these have an FDR < 0.05. Click here for file

Additional file 5 **List of 211 SNPs associated with component of muscle expression with p < 7 × 10^-8 ^in the 32 bimodally expressed genes**. Click here for file

Additional file 6 **Comparison of bimodal gene sets found in the various populations analyzed**. Click here for file
