# Supplementary material for: Bimodal distribution of RNA expression levels in human skeletal muscle tissue
Source: BMC Genomics. 2011 Feb 7;12:98. doi: 10.1186/1471-2164-12-98 (PMC3044673; doi:10.1186/1471-2164-12-98)
Supplement: Additional file 1 — Figure illustrating how dichotomous gene level RNA expression can be artifact of batch effect. [file 1471-2164-12-98-S1.DOC]

**
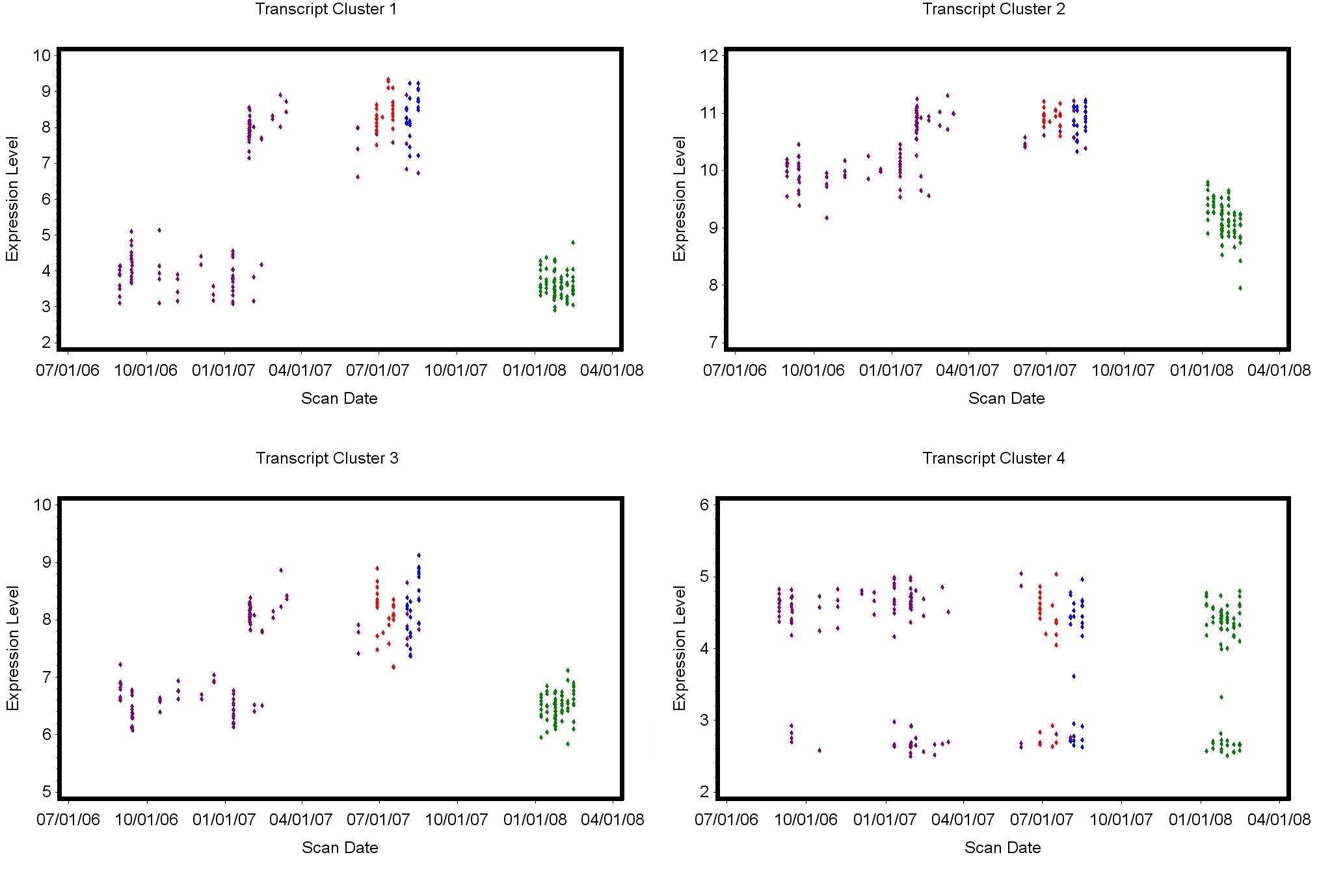
**

**Additional file 1** Dichotomous gene level RNA expression can be artifact of batch effect. Shown are GC-RMA normalized RNA expression levels versus scan date for 4 different transcript clusters (genes) in the 225 chips. Color denotes same lot number (red = I, blue = II, green = III, and purple = Other/Unknown). Different fluidics station sets were used in some timeframes as well. The overall expression level distribution for each of these genes appears multi-modally distributed, artificially due to the batch effect for transcript clusters 1-3, and genuinely for transcript cluster 4 (this example showing bimodal differences associated with gender)
