# Supplementary material for: Bimodal distribution of RNA expression levels in human skeletal muscle tissue
Source: BMC Genomics. 2011 Feb 7;12:98. doi: 10.1186/1471-2164-12-98 (PMC3044673; doi:10.1186/1471-2164-12-98)
Supplement: Additional file 2 — Summary of 16 genes (associated with gender) which met all criteria for bimodality in either group A or group B. [file 1471-2164-12-98-S2.DOC]

| Transcript Number | Chromosome | Gene Symbol | Group A p-value Bimodal N=71 | Group B  p-value Bimodal N=47 | Combined  p-value for Groups A&B | All Chips  p-value Bimodal N=225 | Meets Bimodal Criterion in Chip Group(s) |
| --- | --- | --- | --- | --- | --- | --- | --- |
| 4031136 | Y | *EIF1AY* | 1.51E-48 | 8.52E-29 | 2.26E-74 | 1.3E-111 | A,B,All |
| 4030162 | Y | *DDX3Y* | 7.66E-38 | 4.53E-32 | 5.50E-67 | 3.1E-124 | A,B,All |
| 4035017 | Y | *UTY* | 3E-32 | 2.08E-23 | 7.85E-53 | 1.8E-117 | A,B,All |
| 4030063 | Y | *USP9Y* | 7.21E-30 | 7.21E-15 | 5.23E-42 | 7.96E-96 | A,B,All |
| 4028512 | Y | *RPS4Y1* | 4.52E-28 | 1.3E-30 | 7.80E-56 | 2.0E-117 | A,B,All |
| 4028568 | Y | *ZFY* | 1.36E-26 | 5.84E-13 | 7.05E-37 | 2.64E-78 | A,B,All |
| 4031068 | Y | *CYorf15B* | 7.69E-18 | 5.78E-08 | 2.54E-23 | 3.5E-64 | A,B,All |
| 4036155 | Y | *TTTY10* | 2.93E-08 | 1.39E-04 | 1.11E-10 | 1.6E-28 | A,B,All |
| 2657546 | 3 | *TPRG1* | 1.18E-07 |  |  |  | A |
| 4035833 | Y | *CD24* | 2.19E-06 | 4.86E-04 | 2.31E-08 | 3.7E-23 | A,B,All |
| 3774635 | 17 | *FASN* | 9.83E-06 |  |  |  | A |
| 2374544 | 1 | *IGFN1* | 1.51E-05 |  |  |  | A |
| 3541383 | 14 | *ARG2* | 3.43E-05 | 4.53E-04 | 2.95E-07 | 4.61E-11 | A,B,All |
| 3975467 | X | *UTX* | 6.85E-04 |  |  |  | A |
| 3036924 | 7 | *ACTB* |  | 5.08E-07 |  |  | B |
| 3652218 | 16 | *UQCRC2* |  | 9.59E-06 |  |  | B |

**Additional file 2** Table of 16 genes (associated with gender) which met all criteria for bimodality in either group A or group B. The p-value for bimodality for the entire study (all chips) is shown when all criteria for bimodality were met in this combined group. Blank cells indicate that at least one bimodal criterion of the blank cells for the given gene and data set was not met.
