# Supplementary material for: Bimodal distribution of RNA expression levels in human skeletal muscle tissue
Source: BMC Genomics. 2011 Feb 7;12:98. doi: 10.1186/1471-2164-12-98 (PMC3044673; doi:10.1186/1471-2164-12-98)
Supplement: Additional file 3 — Summary of bimodal genes found on trimmed bimodal data sets of non-gender associated genes. Listed are 28 genes which had a false discovery rate on the Fisher combined p-value from the two trimmed data sets <1.0. Fourteen of these have an FDR < 0.05. [file 1471-2164-12-98-S3.DOC]

| Transcript Number | Chromosome | Gene Symbol | FDR  p-value on Fisher’s p-value | Fisher’s  p-value on Combined Trimmed Groups A&B | Trimmed Group A (n=65)  p-value for Bimodality with 6 d.f. | Trimmed Group B (n=43)  p-value for Bimodality with 6 d.f. | Best cis-SNP pvalue | # SNPsw/in 200kb | p-value  Gender |
| --- | --- | --- | --- | --- | --- | --- | --- | --- | --- |
| 2350981 | 1 | *GSTM1* | 4.41E-40 | 3.54E-44 | 1.96E-25 | 1.71E-21 | **1.20E-11** | 36 | 0.808 |
| 2821347 | 5 | *ERAP2* | 5.46E-14 | 8.76E-18 | 1.89E-13 | 1.05E-06 | **<1E-16** | 94 | 0.943 |
| 4048265 | 6 | *HLA-DRB1* | 3.46E-11 | 8.33E-15 | 9.33E-12 | 2.41E-05 | **<1E-16** | 119 | 0.822 |
| 3336324 | 11 | *ACTN3* | 9.91E-07 | 3.41E-10 | 3.19E-08 | 4.11E-04 | **<1E-16** | 9 | 0.275 |
| 3975227 | X | *MAOA* | 9.91E-07 | 3.97E-10 | 2.25E-07 | 6.81E-05 | **<1E-16** | 69 | 0.537 |
| 4048241 | 6 | *HLA-DRB5* | 2.49E-06 | 1.20E-09 | 2.63E-06 | 1.84E-05 | **2.12E-14** | 104 | 0.907 |
| 2582124 | 2 | *NR4A2* | 5.72E-05 | 3.21E-08 | 9.13E-07 | 1.65E-03 | 0.26 | 36 | 0.171 |
| 2948887 | 6 | *HLA-C* | 3.15E-04 | 2.02E-07 | 1.86E-04 | 5.60E-05 | **<1E-16** | 153 | 0.929 |
| 2418570 | 1 | *SLC44A5* | 2.43E-03 | 1.76E-06 | 1.38E-06 | 0.0743 | **<1E-16** | 112 | 0.954 |
| 3181976 | 9 | *NR4A3* | 8.27E-03 | 6.63E-06 | 9.83E-05 | 4.30E-03 | 0.522 | 20 | 0.401 |
| 2324616 | 1 | *HSPC157* | 0.0128 | 1.13E-05 | 0.0404 | 1.85E-05 | **<1E-16** | 64 | 0.0621 |
| 2492783 | 2 | *THNSL2* | 0.0148 | 1.42E-05 | 4.02E-03 | 2.39E-04 | **<1E-16** | 74 | 0.372 |
| 3682182 | 16 | *ABCC6* | 0.0358 | 3.73E-05 | 8.68E-06 | 0.311 | **2.88E-10** | 59 | 0.953 |
| 3824153 | 19 | *C19orf62* | 0.0499 | 5.60E-05 | 0.0516 | 8.12E-05 | 0.022 | 41 | 0.367 |
| 3618333 | 15 | *MEIS2* | 0.0854 | 1.03E-04 | 4.74E-05 | 0.170 | 0.04 | 86 | 0.995 |
| 3103494 | 8 | *TMEM70* | 0.138 | 1.79E-04 | 3.05E-05 | 0.484 | **<1E-16** | 76 | 0.382 |
| 3680953 | 16 | *FLJ11151* | 0.138 | 1.88E-04 | 4.50E-04 | 0.0346 | **8.62E-7** | 181 | 0.594 |
| 2663295 | 3 | *TMEM40* | 0.269 | 4.08E-04 | 4.75E-05 | 0.765 | 0.000722 | 63 | 0.695 |
| 3836266 | 19 | *FOSB* | 0.269 | 4.11E-04 | 8.86E-03 | 4.13E-03 | 0.00691 | 28 | 0.195 |
| 3332465 | 11 | *MS4A8B* | 0.292 | 4.68E-04 | 4.99E-03 | 8.47E-03 | **<1E-16** | 69 | 0.182 |
| 4037638 | M | *LOC440552* | 0.344 | 5.79E-04 | 3.78E-03 | 0.0141 | **<1E-16** | 8 | 0.853 |
| 2674138 | 3 | *CCDC71* | 0.359 | 6.33E-04 | 1.95E-04 | 0.303 | 0.0468 | 1 | 0.529 |
| 2540007 | 2 | *CYS1* | 0.562 | 1.04E-03 | 1.16E-03 | 0.0880 | 0.00163 | 83 | 0.490 |
| 2344888 | 1 | *CYR61* | 0.619 | 1.19E-03 | 0.0709 | 1.67E-03 | 0.012 | 100 | 0.611 |
| 2830861 | 5 | *EGR1* | 0.736 | 1.48E-03 | 0.0114 | 0.0133 | 0.0356 | 46 | 0.701 |
| 3953911 | 22 | *SLC7A4* | 0.771 | 1.61E-03 | 9.11E-04 | 0.182 | 0.0146 | 32 | 0.995 |
| 3311775 | 10 | *DHX32* | 0.884 | 1.91E-03 | 0.213 | 9.42E-04 | 0.0198 | 105 | 0.135 |
| 3925473 | 21 | *SAMSN1* | 0.956 | 2.15E-03 | 9.91E-03 | 0.0231 | 0.0452 | 110 | 0.963 |

**Additional file 3.** Table of bimodal genes found on trimmed bimodal data sets of non-gender associated genes (N=12,470). Listed are 28 genes which had a false discovery rate on the Fisher’s combined p-value from the two trimmed data sets < 1.0. Fourteen of these have an FDR <0.05. The trimmed data sets had the lowest and highest 5% of expression values deleted for each gene, resulting in Trimmed Group A (n=65) and Trimmed Group B (n=43). The bimodal p-values for each of the data sets come from a chi-square distribution with 6 degrees of freedom. When 3 degrees of freedom are used, a total of 90 genes are found to have a final FDR p-value < 0.05. No limitations based on misclassification area or on the number of data points within each mode were used.
