# Supplementary material for: Bimodal distribution of RNA expression levels in human skeletal muscle tissue
Source: BMC Genomics. 2011 Feb 7;12:98. doi: 10.1186/1471-2164-12-98 (PMC3044673; doi:10.1186/1471-2164-12-98)
Supplement: Additional file 4 — Summary of bimodal genes found on trimmed bimodal data sets of gender associated (p < 0.05) genes. Listed are 30 genes which had a false discovery rate on the Fisher combined p-value from the two trimmed data sets <1.0. Twelve of these have an FDR < 0.05. [file 1471-2164-12-98-S4.DOC]

| Transcript Number | Chr. | Gene Symbol | FDR  p-value on Fisher’s p-value | Fisher’s  p-value on Combined Trimmed Groups A&B | Trimmed Group A (n=65)  p-value for Bimodality with 6 d.f. | Trimmed Group B (n=43)  p-value for Bimodality with 6 d.f. | p-value  Gender |
| --- | --- | --- | --- | --- | --- | --- | --- |
| 4030162 | Y | *DDX3Y* | 4.25E-50 | 7.86E-54 | 6.59E-37 | 9.31E-20 | <1.0E-54 |
| 4031136 | Y | *EIF1AY* | 1.06E-45 | 3.91E-49 | 1.96E-30 | 1.70E-21 | <1.0E-54 |
| 4028512 | Y | *RPS4Y1* | 1.35E-41 | 7.50E-45 | 1.10E-25 | 6.37E-22 | <1.0E-54 |
| 4035017 | Y | *UTY* | 1.56E-39 | 1.15E-42 | 1.35E-29 | 8.33E-16 | <1.0E-54 |
| 4030063 | Y | *USP9Y* | 1.58E-29 | 1.46E-32 | 6.97E-24 | 2.66E-11 | <1.0E-54 |
| 4028568 | Y | *ZFY* | 5.42E-19 | 6.01E-22 | 1.18E-15 | 9.48E-09 | <1.0E-54 |
| 4031068 | Y | *CYorf15B* | 7.35E-18 | 9.51E-21 | 2.91E-14 | 6.40E-09 | 2.20E-42 |
| 3036924 | 7 | *ACTB* | 1.31E-11 | 1.94E-14 | 9.87E-12 | 5.43E-05 | 9.15E-03 |
| 4036155 | Y | *TTTY10* | 8.90E-09 | 1.48E-11 | 9.90E-07 | 5.10E-07 | 5.06E-28 |
| 4035833 | Y | *CD24* | 5.40E-05 | 9.99E-08 | 3.16E-06 | 1.57E-03 | 2.70E-22 |
| 3541383 | 14 | *ARG2* | 6.04E-04 | 1.23E-06 | 4.76E-04 | 1.48E-04 | 0.0241 |
| 2657546 | 3 | *TPRG1* | 9.47E-04 | 2.10E-06 | 5.49E-06 | 0.0226 | 7.77E-31 |
| 2374544 | 1 | *IGFN1* | 0.0820 | 1.97E-04 | 1.73E-05 | 0.945 | 0.0422 |
| 4024685 | X | *SLITRK4* | 0.0858 | 2.22E-04 | 1.15E-03 | 0.0163 | 5.09E-04 |
| 3745161 | 17 | *MYH8* | 0.137 | 3.79E-04 | 3.71E-03 | 9.04E-03 | 0.0286 |
| 3975467 | X | *UTX* | 0.163 | 4.82E-04 | 1.51E-04 | 0.289 | 3.77E-24 |
| 3915936 | 21 | *NCAM2* | 0.181 | 5.68E-04 | 2.11E-03 | 0.0248 | 0.0121 |
| 3751058 | 17 | *C17orf63* | 0.185 | 6.16E-04 | 0.890 | 6.43E-05 | 1.32E-07 |
| 3774635 | 17 | *FASN* | 0.390 | 1.37E-03 | 1.22E-03 | 0.114 | 7.64E-03 |
| 3432641 | 12 | *C12orf52* | 0.428 | 1.58E-03 | 1.98E-04 | 0.820 | 0.0150 |
| 3452231 | 12 | *SLC38A1* | 0.544 | 2.13E-03 | 0.169 | 1.34E-03 | 0.0218 |
| 3756613 | 17 | *KRT39* | 0.544 | 2.21E-03 | 3.47E-04 | 0.683 | 0.0157 |
| 3869062 | 19 | *SIGLEC8* | 0.632 | 2.68E-03 | 2.99E-04 | 0.983 | 0.0160 |
| 3963676 | 22 | *C22orf9* | 0.661 | 2.93E-03 | 4.70E-03 | 0.0690 | 0.0462 |
| 3309755 | 10 | *C10orf119* | 0.669 | 3.19E-03 | 1.00 | 3.57E-04 | 9.70E-03 |
| 2428119 | 1 | *KCND3* | 0.669 | 3.22E-03 | 0.111 | 3.24E-03 | 7.90E-03 |
| 3744324 | 17 | *C17orf68* | 0.760 | 3.79E-03 | 4.50E-03 | 0.0964 | 0.0268 |
| 3811596 | 18 | *SERPINB3* | 0.895 | 4.63E-03 | 0.0248 | 0.0219 | 4.72E-03 |
| 3457101 | 12 | *ITGA7* | 0.958 | 5.24E-03 | 0.851 | 7.35E-04 | 0.0129 |
| 2634058 | 3 | *FAM55C* | 0.958 | 5.31E-03 | 4.51E-03 | 0.141 | 4.98E-03 |

**Additional file 4** Summary of bimodal genes found on trimmed bimodal data sets of gender associated (p<0.05) genes (N=5,411). Listed are 30 genes which had a false discovery rate on the Fisher combined p-value from the two trimmed data sets < 1.0. Twelve of these have an FDR <0.05. The trimmed data sets had the lowest and highest 5% of expression values deleted for each gene, resulting in Trimmed Group A (n=65) and Trimmed Group B (n=43). The bimodal p-values for each of the data sets come from a chi-square distribution with 6 degrees of freedom. When 3 degrees of freedom are used, a total of 60 genes are found to have a final FDR p-value < 0.05. No limitations based on misclassification area or on the number of data points within each component were used.
