# Supplementary material for: Bimodal distribution of RNA expression levels in human skeletal muscle tissue
Source: BMC Genomics. 2011 Feb 7;12:98. doi: 10.1186/1471-2164-12-98 (PMC3044673; doi:10.1186/1471-2164-12-98)
Supplement: Additional file 5 — List of 211 SNPs associated with component of muscle expression with p < 7 × 10^-8^ in the 32 bimodally expressed genes. [file 1471-2164-12-98-S5.DOC]

| SNP rs Number | Gene Symbol | Chr. | p-val |
| --- | --- | --- | --- |
| rs11807 | GSTM1 | 1 | 1.20E-11 |
| rs1887546 | GSTM1 | 1 | 1.93E-08 |
| rs2094469 | GSTM1 | 1 | 5.87E-08 |
| rs35369793 | HLA-DRB1 | 6 | <1E-16 |
| rs9270986 | HLA-DRB1 | 6 | <1E-16 |
| rs9268877 | HLA-DRB1 | 6 | 5.55E-16 |
| rs9268541 | HLA-DRB1 | 6 | 5.66E-15 |
| rs3135392 | HLA-DRB1 | 6 | 2.52E-10 |
| rs7766843 | HLA-DRB1 | 6 | 1.01E-08 |
| rs7766843 | HLA-DRB1 | 6 | 1.01E-08 |
| rs9268856 | HLA-DRB1 | 6 | 1.01E-08 |
| rs9268861 | HLA-DRB1 | 6 | 1.01E-08 |
| rs9268862 | HLA-DRB1 | 6 | 1.01E-08 |
| rs9268878 | HLA-DRB1 | 6 | 1.01E-08 |
| rs7756262 | HLA-DRB1 | 6 | 2.88E-08 |
| rs9268557 | HLA-DRB1 | 6 | 2.88E-08 |
| rs615672 | HLA-DRB1 | 6 | 2.98E-08 |
| rs9268542 | HLA-DRB1 | 6 | 3.06E-08 |
| rs9268645 | HLA-DRB1 | 6 | 4.67E-08 |
| rs9268832 | HLA-DRB1 | 6 | 4.91E-08 |
| rs9268560 | HLA-DRB1 | 6 | 5.04E-08 |
| rs10434709 | ERAP2 | 5 | <1E-16 |
| rs13167902 | ERAP2 | 5 | <1E-16 |
| rs18059 | ERAP2 | 5 | <1E-16 |
| rs1974871 | ERAP2 | 5 | <1E-16 |
| rs2548533 | ERAP2 | 5 | <1E-16 |
| rs2549794 | ERAP2 | 5 | <1E-16 |
| rs2549797 | ERAP2 | 5 | <1E-16 |
| rs27300 | ERAP2 | 5 | <1E-16 |
| rs27307 | ERAP2 | 5 | <1E-16 |
| rs27397 | ERAP2 | 5 | <1E-16 |
| rs27711 | ERAP2 | 5 | <1E-16 |
| rs27712 | ERAP2 | 5 | <1E-16 |
| rs2910787 | ERAP2 | 5 | <1E-16 |
| rs3909451 | ERAP2 | 5 | <1E-16 |
| rs9127 | ERAP2 | 5 | <1E-16 |
| rs27290 | ERAP2 | 5 | 1.11E-16 |
| rs2278019 | ERAP2 | 5 | 2.22E-16 |
| rs11135482 | ERAP2 | 5 | 8.44E-14 |
| rs27993 | ERAP2 | 5 | 1.52E-13 |
| rs6861399 | ERAP2 | 5 | 2.64E-11 |
| rs1230358 | ERAP2 | 5 | 2.83E-11 |
| rs13165925 | ERAP2 | 5 | 3.23E-11 |
| rs11748432 | ERAP2 | 5 | 8.55E-11 |
| rs10043135 | ERAP2 | 5 | 9.40E-10 |
| rs2544776 | ERAP2 | 5 | 9.40E-10 |
| rs316224 | ERAP2 | 5 | 9.40E-10 |
| rs316234 | ERAP2 | 5 | 1.90E-09 |
| rs2549779 | ERAP2 | 5 | 8.50E-09 |
| rs35369793 | HLA-DRB5 | 6 | 2.12E-14 |
| rs9270986 | HLA-DRB5 | 6 | 1.34E-12 |
| rs7747521 | HLA-DRB5 | 6 | 3.09E-12 |
| rs7766843 | HLA-DRB5 | 6 | 6.58E-12 |
| rs7766843 | HLA-DRB5 | 6 | 6.58E-12 |
| rs9268856 | HLA-DRB5 | 6 | 6.58E-12 |
| rs9268861 | HLA-DRB5 | 6 | 6.58E-12 |
| rs9268862 | HLA-DRB5 | 6 | 6.58E-12 |
| rs9268878 | HLA-DRB5 | 6 | 6.58E-12 |
| rs3135392 | HLA-DRB5 | 6 | 7.77E-12 |
| rs615672 | HLA-DRB5 | 6 | 2.98E-11 |
| rs9268877 | HLA-DRB5 | 6 | 5.71E-11 |
| rs502771 | HLA-DRB5 | 6 | 1.19E-10 |
| rs674313 | HLA-DRB5 | 6 | 1.38E-10 |
| rs5000563 | HLA-DRB5 | 6 | 1.53E-10 |
| rs9268832 | HLA-DRB5 | 6 | 1.64E-10 |
| rs7756262 | HLA-DRB5 | 6 | 1.71E-10 |
| rs9268557 | HLA-DRB5 | 6 | 1.71E-10 |
| rs7746922 | HLA-DRB5 | 6 | 1.80E-10 |
| rs9268560 | HLA-DRB5 | 6 | 2.91E-10 |
| rs9268645 | HLA-DRB5 | 6 | 2.93E-10 |
| rs6930933 | HLA-DRB5 | 6 | 3.65E-10 |
| rs642093 | HLA-DRB5 | 6 | 3.82E-10 |
| rs7194 | HLA-DRB5 | 6 | 3.94E-10 |
| rs2395161 | HLA-DRB5 | 6 | 3.97E-10 |
| rs2001097 | HLA-DRB5 | 6 | 4.14E-10 |
| rs2213580 | HLA-DRB5 | 6 | 4.14E-10 |
| rs2395164 | HLA-DRB5 | 6 | 4.14E-10 |
| rs3129872 | HLA-DRB5 | 6 | 4.14E-10 |
| rs3135366 | HLA-DRB5 | 6 | 4.14E-10 |
| rs3135378 | HLA-DRB5 | 6 | 4.14E-10 |
| rs3135393 | HLA-DRB5 | 6 | 4.14E-10 |
| rs4988822 | HLA-DRB5 | 6 | 4.14E-10 |
| rs9268542 | HLA-DRB5 | 6 | 4.19E-10 |
| rs9268541 | HLA-DRB5 | 6 | 4.67E-10 |
| rs3135363 | HLA-DRB5 | 6 | 4.74E-10 |
| rs7195 | HLA-DRB5 | 6 | 5.05E-10 |
| rs2187668 | HLA-DRB5 | 6 | 5.74E-10 |
| rs3135342 | HLA-DRB5 | 6 | 6.74E-10 |
| rs2395167 | HLA-DRB5 | 6 | 8.13E-10 |
| rs2001099 | HLA-DRB5 | 6 | 9.50E-10 |
| rs3129877 | HLA-DRB5 | 6 | 1.02E-09 |
| rs3129878 | HLA-DRB5 | 6 | 1.51E-09 |
| rs3135376 | HLA-DRB5 | 6 | 1.64E-09 |
| rs1051336 | HLA-DRB5 | 6 | 2.99E-09 |
| rs3129847 | HLA-DRB5 | 6 | 3.33E-09 |
| rs3135376 | HLA-DRB5 | 6 | 5.40E-09 |
| rs7194 | HLA-DRB5 | 6 | 2.14E-08 |
| rs2205718 | MAOA | 23 | <1E-16 |
| rs2235185 | MAOA | 23 | <1E-16 |
| rs2235186 | MAOA | 23 | <1E-16 |
| rs3027401 | MAOA | 23 | <1E-16 |
| rs3788863 | MAOA | 23 | <1E-16 |
| rs5905859 | MAOA | 23 | <1E-16 |
| rs5906883 | MAOA | 23 | <1E-16 |
| rs5953385 | MAOA | 23 | <1E-16 |
| rs6323 | MAOA | 23 | <1E-16 |
| rs909525 | MAOA | 23 | <1E-16 |
| rs979605 | MAOA | 23 | <1E-16 |
| rs979606 | MAOA | 23 | <1E-16 |
| rs2064070 | MAOA | 23 | 2.02E-14 |
| rs6609257 | MAOA | 23 | 5.38E-14 |
| rs509556 | ACTN3 | 11 | <1E-16 |
| rs540874 | ACTN3 | 11 | <1E-16 |
| rs572846 | ACTN3 | 11 | <1E-16 |
| rs580910 | ACTN3 | 11 | <1E-16 |
| rs470300 | ACTN3 | 11 | 7.90E-14 |
| rs2242663 | ACTN3 | 11 | 3.43E-10 |
| rs1791687 | ACTN3 | 11 | 5.83E-09 |
| rs615536 | ACTN3 | 11 | 9.21E-09 |
| rs1053054 | TMEM70 | 8 | <1E-16 |
| rs7843858 | TMEM70 | 8 | <1E-16 |
| rs12547746 | TMEM70 | 8 | 1.83E-10 |
| rs4738407 | TMEM70 | 8 | 7.40E-10 |
| rs7839013 | TMEM70 | 8 | 7.40E-10 |
| rs1249657 | SLC44A5 | 1 | <1E-16 |
| rs1249811 | SLC44A5 | 1 | <1E-16 |
| rs1343519 | SLC44A5 | 1 | <1E-16 |
| rs2347218 | SLC44A5 | 1 | <1E-16 |
| rs2587031 | SLC44A5 | 1 | <1E-16 |
| rs1953077 | SLC44A5 | 1 | 7.39E-13 |
| rs6670304 | SLC44A5 | 1 | 1.11E-12 |
| rs2057519 | SLC44A5 | 1 | 2.07E-10 |
| rs2657206 | SLC44A5 | 1 | 7.88E-10 |
| rs10493573 | SLC44A5 | 1 | 8.27E-10 |
| rs11163089 | SLC44A5 | 1 | 8.27E-10 |
| rs12404058 | SLC44A5 | 1 | 8.27E-10 |
| rs10493571 | SLC44A5 | 1 | 1.01E-09 |
| rs609574 | SLC44A5 | 1 | 3.01E-09 |
| rs696676 | SLC44A5 | 1 | 6.64E-09 |
| rs627494 | SLC44A5 | 1 | 7.94E-09 |
| rs12562924 | SLC44A5 | 1 | 1.19E-08 |
| rs11582321 | SLC44A5 | 1 | 3.93E-08 |
| rs3851722 | ABCC6 | 16 | 2.88E-10 |
| rs212097 | ABCC6 | 16 | 1.37E-09 |
| rs7192634 | ABCC6 | 16 | 7.21E-09 |
| rs169844 | ABCC6 | 16 | 9.80E-09 |
| rs4780599 | ABCC6 | 16 | 1.11E-08 |
| rs212085 | ABCC6 | 16 | 1.46E-08 |
| rs212070 | ABCC6 | 16 | 2.27E-08 |
| rs4577106 | ABCC6 | 16 | 2.46E-08 |
| rs2283508 | ABCC6 | 16 | 3.55E-08 |
| rs212087 | ABCC6 | 16 | 6.73E-08 |
| rs10173112 | THNSL2 | 2 | <1E-16 |
| rs10185660 | THNSL2 | 2 | <1E-16 |
| rs4359651 | THNSL2 | 2 | <1E-16 |
| rs9636487 | THNSL2 | 2 | <1E-16 |
| rs7581571 | THNSL2 | 2 | 1.11E-16 |
| rs12621922 | THNSL2 | 2 | 1.69E-12 |
| rs3130531 | HLA-C | 6 | <1E-16 |
| rs3130952 | HLA-C | 6 | <1E-16 |
| rs3132488 | HLA-C | 6 | <1E-16 |
| rs7382297 | HLA-C | 6 | <1E-16 |
| rs9264523 | HLA-C | 6 | <1E-16 |
| rs2524066 | HLA-C | 6 | 1.11E-16 |
| rs2524095 | HLA-C | 6 | 1.11E-16 |
| rs2524089 | HLA-C | 6 | 2.22E-16 |
| rs3130941 | HLA-C | 6 | 2.22E-16 |
| rs2596509 | HLA-C | 6 | 3.34E-13 |
| rs1811197 | HLA-C | 6 | 1.14E-12 |
| rs3915971 | HLA-C | 6 | 2.53E-12 |
| rs3132496 | HLA-C | 6 | 1.18E-11 |
| rs2524115 | HLA-C | 6 | 1.79E-11 |
| rs28480108 | HLA-C | 6 | 1.81E-11 |
| rs2844615 | HLA-C | 6 | 8.03E-11 |
| rs2853934 | HLA-C | 6 | 8.03E-11 |
| rs2853948 | HLA-C | 6 | 8.03E-11 |
| rs396038 | HLA-C | 6 | 8.03E-11 |
| rs2524051 | HLA-C | 6 | 1.85E-10 |
| rs3873385 | HLA-C | 6 | 2.08E-10 |
| rs3132486 | HLA-C | 6 | 3.29E-10 |
| rs6906846 | HLA-C | 6 | 3.61E-10 |
| rs3130942 | HLA-C | 6 | 4.87E-10 |
| rs2853943 | HLA-C | 6 | 5.32E-10 |
| rs3134745 | HLA-C | 6 | 8.64E-10 |
| rs2523534 | HLA-C | 6 | 1.00E-09 |
| rs5006724 | HLA-C | 6 | 1.25E-09 |
| rs7743761 | HLA-C | 6 | 1.77E-09 |
| rs9501572 | HLA-C | 6 | 1.77E-09 |
| rs4081552 | HLA-C | 6 | 3.84E-09 |
| rs3095238 | HLA-C | 6 | 5.00E-09 |
| rs2524057 | HLA-C | 6 | 5.70E-09 |
| rs3131018 | HLA-C | 6 | 7.59E-09 |
| rs2524123 | HLA-C | 6 | 9.26E-09 |
| rs3131009 | HLA-C | 6 | 1.06E-08 |
| rs1051796 | HLA-C | 6 | 1.11E-08 |
| rs2246954 | HLA-C | 6 | 1.36E-08 |
| rs12110795 | HLA-C | 6 | 2.43E-08 |
| rs11586488 | HSPC157 | 1 | <1E-16 |
| rs2056974 | HSPC157 | 1 | 1.11E-16 |
| rs10917139 | HSPC157 | 1 | 2.22E-16 |
| rs10917145 | HSPC157 | 1 | 3.33E-16 |
| rs16826595 | HSPC157 | 1 | 5.55E-16 |
| rs2072920 | HSPC157 | 1 | 6.99E-15 |
| rs10493011 | HSPC157 | 1 | 1.80E-14 |
| rs17837965 | HSPC157 | 1 | 3.78E-13 |
| rs3856181 | HSPC157 | 1 | 1.36E-09 |
| rs12749135 | HSPC157 | 1 | 2.49E-09 |
| rs2491213 | HSPC157 | 1 | 3.02E-09 |
| rs2505711 | HSPC157 | 1 | 4.32E-09 |
| rs7529773 | HSPC157 | 1 | 9.61E-09 |
| rs732266 | HSPC157 | 1 | 6.83E-08 |

**Additional file 5** List of 211 SNPs associated with component of muscle expression with p < 7x10^-8^ in the 32 bimodally expressed genes listed in Table 3.
