# Supplementary material for: Bimodal distribution of RNA expression levels in human skeletal muscle tissue
Source: BMC Genomics. 2011 Feb 7;12:98. doi: 10.1186/1471-2164-12-98 (PMC3044673; doi:10.1186/1471-2164-12-98)
Supplement: Additional file 6 — Comparison of bimodal gene sets found in the various populations analyzed. [file 1471-2164-12-98-S6.DOC]

| Data Sets I/II | Genes in Common | Bimodals in I | Bimodals in II | Common Bimodals | p-value |
| --- | --- | --- | --- | --- | --- |
| Pima/GSE1485 | 3332 | 10 | 144 | 8 | 4.21E-9 |
| Pima/GSE5086 | 16242 | 25 | 282 | 9 | 2.02E-10 |
| Pima/GSE13070 | 16242 | 25 | 259 | 10 | 2.37E-12 |
| GSE1485/GSE5086 | 3455 | 154 | 70 | 15 | 2.38E-7 |
| GSE1485/GSE13070 | 3455 | 154 | 110 | 10 | 0.0295 |
| GSE5086/GSE13070* | 20309 | 340 | 303 | 20 | 2.16E-7 |
| GSE5086/GSE13070** | 41789 | 360 | 331 | 23 | 2.32E-14 |

**Additional file 6** Comparison of bimodal gene sets found in various populations analyzed. P-values for chance concordance of bimodal gene sets were calculated from Fisher’s exact test [49]. Bimodality in Pimas reflects genes identified as having an FDR <0.05 on the Fisher combined p-value from the trimmed data of the two batches, each with bimodal p-values calculated from a chi-square distribution with 6 degrees of freedom (Table 6). Bimodal genes in other data sets reflect a p-value < 0.001 for a chi-square distribution with 6 degrees of freedom. Genes in common were determined based on gene name and in the non-Pima data sets, using the probe with best evidence of bimodality for a gene when multiple probes occurred for a gene. * comparing best probe per gene ** comparing all probes
